# Global serum profiling: an opportunity for earlier cancer detection

**DOI:** 10.1186/s13046-023-02786-y

**Published:** 2023-08-15

**Authors:** Alexandra Sala, James M. Cameron, Paul M. Brennan, Emma J. Crosbie, Tom Curran, Ewan Gray, Pierre Martin-Hirsch, David S. Palmer, Ihtesham U. Rehman, Nicholas J. W. Rattray, Matthew J. Baker

**Affiliations:** 1 Dxcover Limited, Glasgow, G1 1XW UK; 2 Translational Neurosurgery, Department of Clinical Neurosciences, Royal Infirmary of Edinburgh, Edinburgh, EH16 4SB UK; 3 Division of Cancer Sciences, School of Medical Sciences, Faculty of Biology, Medicine and Health, University of Manchester, Manchester, M13 9PL UK; 4 Division of Gynecology, St Mary’s Hospital, Manchester University NHS Foundation Trust, Manchester, M13 9WL UK; 5 Children’s Mercy Research Institute, Children’s Mercy Kansas City, Kansas City, MO 64108 USA; 6 Independent Health Economics Consultant, Edinburgh, UK; 7 Gynecological Oncology, Clinical Research Facility, Lancashire Teaching Hospitals, Preston, PR2 9HT UK; 8 Department of Pure and Applied Chemistry, Thomas Graham Building, University of Strathclyde, Glasgow, G1 1XL UK; 9 School of Medicine, Faculty of Clinical and Biomedical Sciences, University of Central Lancashire, Preston, PR1 2HE UK; 10 Strathclyde Institute of Pharmacy and Biomedical Sciences (SIPBS), University of Strathclyde, Glasgow, G4 0RE UK

**Keywords:** Liquid biopsy, Blood serum, Cancer detection, Diagnosis, Tumorigenesis, Metabolism, Immune system, Genetics, Spectroscopy, Pan-omics.

## Abstract

The advances in cancer research achieved in the last 50 years have been remarkable and have provided a deeper knowledge of this disease in many of its conceptual and biochemical aspects. From viewing a tumor as a ‘simple’ aggregate of mutant cells and focusing on detecting key cell changes leading to the tumorigenesis, the understanding of cancer has broadened to consider it as a complex organ interacting with its close and far surroundings through tumor and non-tumor cells, metabolic mechanisms, and immune processes. Metabolism and the immune system have been linked to tumorigenesis and malignancy progression along with cancer-specific genetic mutations. However, most technologies developed to overcome the barriers to earlier detection are focused solely on genetic information. The concept of cancer as a complex organ has led to research on other analytical techniques, with the quest of finding a more sensitive and cost-effective comprehensive approach. Furthermore, artificial intelligence has gained broader consensus in the oncology community as a powerful tool with the potential to revolutionize cancer diagnosis for physicians. We herein explore the relevance of the concept of cancer as a complex organ interacting with the bodily surroundings, and focus on promising emerging technologies seeking to diagnose cancer earlier, such as liquid biopsies. We highlight the importance of a comprehensive approach to encompass all the tumor and non-tumor derived information salient to earlier cancer detection.

## Introduction

Cancer research has realized outstanding achievements in the last 50 years with the development of new analytical technologies and genetic knowledge, providing fresh insights in detection, therapeutics, and monitoring of diseases. Notwithstanding the increase in net survival rates, cancer still represents a worldwide burden which has been predicted to increase in incidence of 57% by 2040, followed by a 64% rise in mortality [1–3]. Due to its increasing incidence, cancer has been predicted to overtake cardiovascular diseases as the primary cause of death by 2030, with over 640,000 fatalities every year in the U.S [4]; this changeover is also partly favored by the continuously improving prevention measures and survival rates of cardiovascular diseases.

Researchers have been developing technologies suitable for earlier cancer detection to improve survival rates of patients diagnosed with cancer. Earlier detection could allow an increase in the efficacy of surgical resection and other therapies, and reduce the psychological and economic burden of late-stage cancer diagnosis. The development of technologies that are cost-effective, easy-to-use, and less invasive than tissue biopsy, is essential for the detection of tumors in the pre-cancerous or initial stages of the disease.

Biochemical mechanisms leading to tumorigenesis have been the focus of numerous studies in the past two decades, to further comprehend metabolic processes and the involvement of the immune system during malignant cells’ growth and proliferation [5–9]. Genetic tests have gained interest as tools for detecting mutations and cancer biomarkers through advanced technologies, such as DNA (i.e., deoxy-ribonucleic acid) sequencing, but given the limitations of genetic testing alone, there has recently been extensive research in other analytical fields with the quest of finding a more sensitive and cost-effective ‘pan-omic’ approach [10].

Artificial intelligence (AI) has allowed the discovery of signals previously undetectable by human operators or with traditional statistical methods, and can now support the combination of several analytical results together with known risk factors (e.g., age) and other clinically relevant information (e.g., symptomatology) to improve test classification performance, and hence disease predictions [11–15]. With the improvements in diagnostic accuracy and better knowledge regarding key biomarkers for cancer detection, AI could pave the way for a more efficient decision process to streamline patients for urgent referral (e.g., imaging) or routine follow-up [16].

In this review article, we discuss the concept of tumors as complex organs interacting with all the bodily surroundings and the current biochemical paradigms of tumorigenesis and cancer progression, which have been extensively discussed by Hanahan, Pavlova, and others [5–9, 17], to provide the reader with a general knowledge on the biochemical events occurring during neoplasia formation. Subsequently, we introduce the importance of pan-omic technologies to encompass the complex nature of the tumor microenvironment (TME) and its interaction with the human body. We also highlight the benefits of liquid biopsy techniques to incorporate all tumor and non-tumor derived information needed for earlier cancer detection. To conclude, we discuss the concept of combination testing involving currently developed technologies, as a potential strategy for a combined early detection liquid biopsy.

## Oncogenesis

Cancer is a complex organ [18]. Tumors are aggregates of multiple cell types interfacing with the entire organism through their microenvironment. A long-held paradigm has been to consider cancer as solely a genetic disease, given the complexity of genetic changes that are associated with tumorigenesis [19]. Although, tumors behave as a much more complex system, incorporating a variety of non-tumor derived cells, not only genetically altered tumor cells [18]. Certain characteristics of oncogenesis resemble processes of both organ development and tissue remodeling; especially the TME, which undergoes favorable surrounding changes for cell growth and proliferation [20]. Technologies capable of exploiting all features of the complex interaction between cancer cells and the whole body, locally and systemically including mutational, metabolic, and immune responses, could be key to achieving the early detection of cancer.

To better comprehend the factors involved in tumor formation (i.e., oncogenesis), it is imperative to examine the ‘hallmarks of cancer’; these can be defined as those principles that together form an organized framework of significant characteristics apt to describe the mechanisms and processes contributing to neoplastic formation on both a genetic and metabolic level [6, 9]. Nonetheless, the immune system interaction in cancer formation is thought to be an ‘emerging hallmark’, playing a significant role in promoting an inflammatory state which may encourage, but may also inhibit, the formation of neoplasia [6, 21]. The mechanisms that occur in the human body surrounding the TME are still not fully understood, presenting many challenges in the quest for early detection and successful therapies [22–24].

### The hallmarks of cancer

**Fig. 1:**
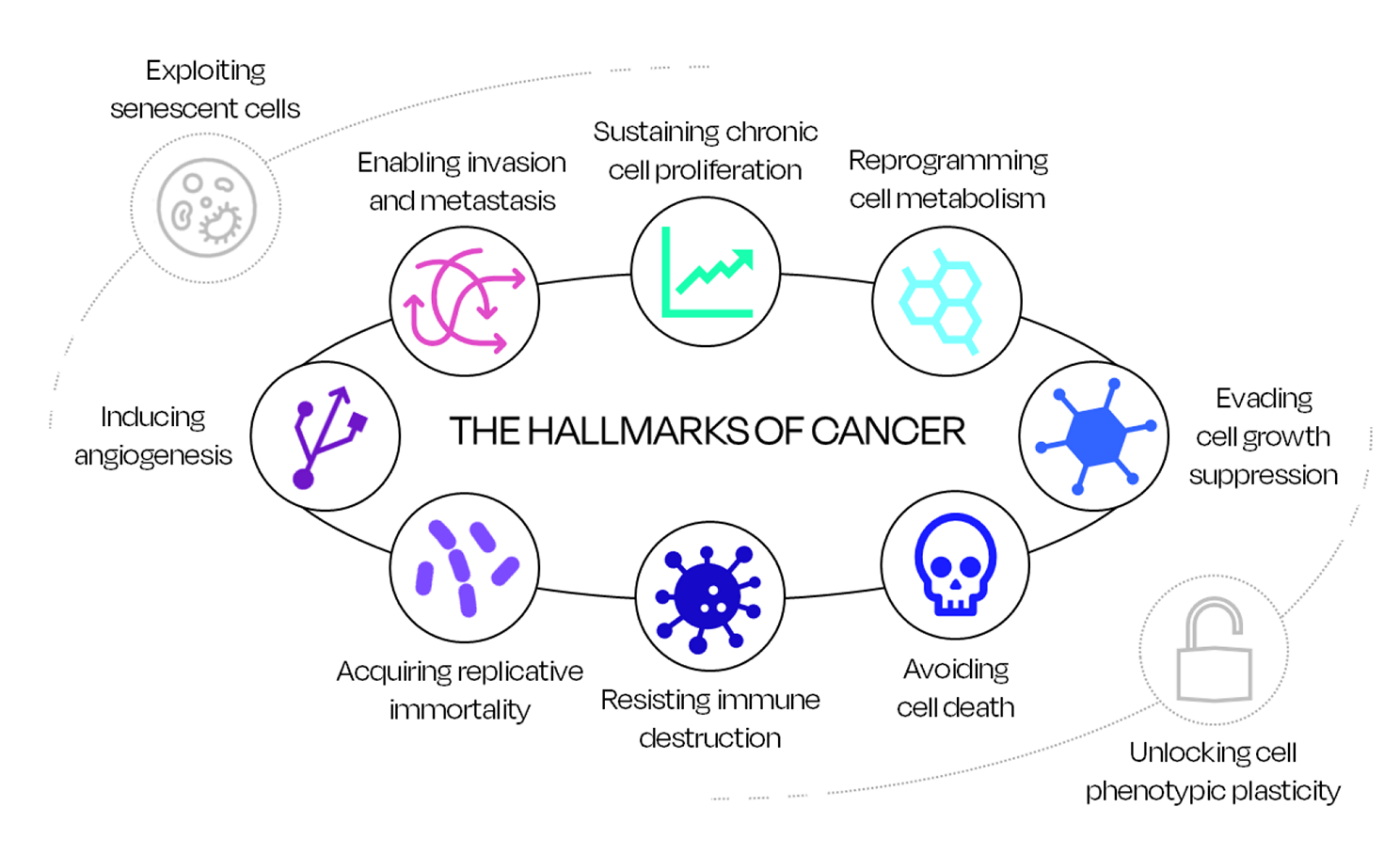
The hallmarks of cancer according to Hanahan and Weinberg [5–7]. The eight inner circles identify the confirmed hallmarks, whilst the two outer circles consist of the additional emerging hallmarks

Hanahan and Weinberg first proposed six hallmarks of cancer in 2000, and in 2011 added two new emerging hallmarks [5, 6]. In 2022, Hanahan corroborated the eight hallmarks and proposed two additional emerging hallmarks, which are described in the text below [7]. Figure 1 schematically incorporates all the ten hallmarks of cancer (eight confirmed and two additional proposed).

#### Sustaining chronic cell proliferation

The cardinal attribute of cancer consists of sustaining chronic proliferation of tumor cells. Activating signals are typically transmitted by growth factor ligands binding cell-surface receptors, through intracellular signaling pathways, which regulate the cell cycle and cell development, survival, and metabolism. Despite the complicated nature of these signals in both normal and cancer tissues, the mechanism of proliferative signaling in the TME have been thoroughly investigated and understood [6]; cancer cells can self-produce growth factors ligands or stimulate normal cells to receive growth factors supplies [25–28].

#### Reprogramming cell metabolism

Adjustments of energy metabolism are necessary to sustain the uncontrolled tumor cell proliferation, as cells need to be fueled with energy to undergo growth-and-division cycles. Normal cells undergo glycolysis in aerobic conditions to produce a high yield of adenosine triphosphate (ATP; i.e., metabolic energy); in anaerobic conditions, glycolysis can still happen, however the energy yield is lower. Tumor cells systematically use anaerobic glycolysis, resulting in a consistently increased glucose uptake to compensate for the reduced ATP production; this characteristic of cancer cells metabolism takes the name of ‘Warburg’s effect’ [6, 29]. Other tumor cells use lactate (i.e., by-product of anaerobic glycolysis) as their main source of energy, creating a perfectly functioning symbiotic system [30]. In addition to glucose and lactate, amino acids have also been recently claimed as significant opportunistic fuel sources for cancer development; glutamine and branched-chain amino acids (BCAA) specifically contribute to the support of the acid citric cycle (or tricarboxylic acid, TCA, cycle) [31].

#### Evading cell growth suppression

Tumor suppressor genes typically encode retinoblastoma-associated protein (RB) and tumor protein 53 (TP53), which control both cell proliferation, and activation of senescence (i.e., dormant state) and apoptosis (i.e., cell death) [6]. RB is a gate-keeper in the cell-cycle and defects in the protein can result in persistent proliferation [32]. In the same way, TP53 helps with regulatory circuits by halting the cell-cycle progression when stress/abnormality sensors are signaling an elevated level of genomic damage; its deficiency can allow the cancer cell to progress through its growth [6]. Furthermore, corruption of the anti-proliferative transforming grow factor β (TGF-β) pathway contribute to cell growth, and is believed to be key in the development of high-grade malignancy associated cell traits [6, 33].

#### Avoiding cell death

Genomic damage halts the cell-cycle to repair the altered sequence or send signals of activating cell death when the damage is too extensive. Some cancer cells can resist this natural barrier, avoiding programmed cell death by apoptosis. However, not all cells are immune to apoptosis; many cancer cells still experience cell death due to the physiological stress caused by tumorigenesis and/or anticancer therapy [6]. Cytochrome c, Bax and Bak are the regulatory proteins of the Bcl-2 family responsible to counterbalance the pro- and anti-apoptotic signals; damages to the transcription of these proteins will cause unbalances in the regulatory processes of the cells [34]. Autophagy and necrosis are also triggered by genomic stress/damage [35–37].

#### Resisting immune destruction

Not all immune cells are tumor promoting, most immune system cells are apt to defend the body from pathogens, and cancer cells are often seen as one of them, activating the immune cascade [6]. Originally introduced as an emerging hallmark, the ability of cancer cells to actively avoid immune system elimination has been confirmed by Hanahan, and numerous experiments have been conducted to investigate this hallmark [6, 7]. The importance of the immune system in fighting cancer formation and proliferation has been studied by comparing immunocompromised subjects and transplant carriers, showing how cancers form more easily in weak immunogenic environments [38–40].

#### Acquiring replicative immortality

The ability of cancer cells to acquire replicative immortality can be described as the capacity of undergoing a sufficient number of successive growth-and-division cell cycles to generate macroscopic tumors [6]. Telomeres, which are specific DNA-protein structures found at the ends of the chromosomes and protect their genomic sequence from various negative replication events, are crucial for this hallmark [41]. Their length dictates the quantity of growth-and-division cycles that a cell can sustain, hence replicative immortality is enabled by variant cells that maintain telomeric DNA at sufficient lengths avoiding the senescence/apoptosis trigger [6].

#### Inducing angiogenesis

During neoplastic growth, new blood vessels sprout from existing ones (i.e., angiogenesis) to facilitate tumor expansion, constituting an established ‘angiogenic switch’ that seems to be only activated and maintained during tumorigenesis [42]. There are a variety of factors that are considered proangiogenic and contribute to the activation of the angiogenic switch. A well-known angiogenic inducer is the vascular endothelial growth factor-A (VEGF-A) gene, which encodes ligands apt to the growth of new vessels in several stages of the individual progression and health maintenance, from favoring the embryonic development to counteracting adult pathological situations [6]. Oncogene signaling upregulates VEGF gene expression [43].

#### Enabling invasion and metastasis

**Fig. 2:**
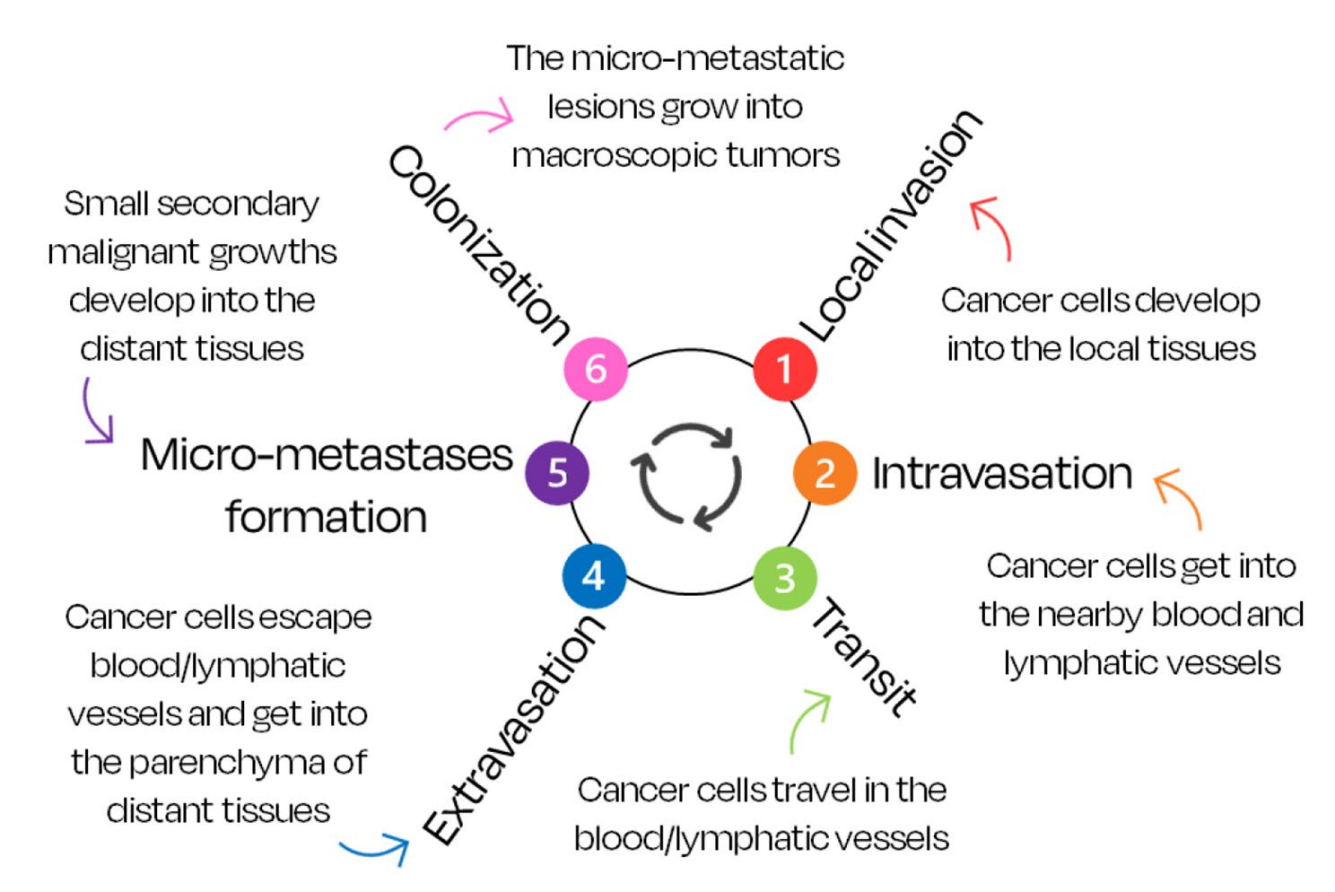
The ‘invasion-metastasis’ cascade [5–7, 46, 47]

Once the primary tumor has formed, the multi-step process of invasion and metastasis occurs as described in Fig. 2. The combination of the processes that lead to invasion of tumor cells, and metastases formation and colonization is complex, hence there are many hypotheses regarding their functioning and importance [6]. It has been shown that some cancers can metastasize in earlier stages of the disease; micro-metastases can disperse from tumors with non-invasive nature and the poor capacity to hold cancer cells in their blood/lymphatic systems results in easier invasion of the parenchyma [44, 45].

#### Additional emerging hallmark: unlocking cell phenotypic plasticity

Phenotypic plasticity refers to the ability of the cells to modify their behavioral response based on the environmental impulses. Cell plasticity is usually well constructed, and cells follow a prefixed path from cell of origin to a terminal differentiated cell, in which a cell changes to specialize for a specific function. The ability of a cancerous state to unlock this phenotypic plasticity results in a deregulated cell differentiation where they evade the preordained differentiation [7, 48]. The alterations of the differentiation pathway are found in various cancer types during the primary tumor formation, the development to malignancy and, in some cases, in response to therapeutics [7].

#### Additional emerging hallmark: exploiting senescent cells

Hanahan and Weinberg have described senescence in relation to the ability of cancer cells to escape the senescence stage and enable replicative immortality [6]. The senescence-associated secretory phenotype (SASP) components include growth and pro-inflammatory factors, such as cytokines, among a variety of enzymes and proteins [49]. SASP is responsible of senescent cells secreting higher levels of growth promoting factors, which trigger the formation of neoplasia through conveying access to also other important hallmarks; such as continuous proliferation, escaping cell death, favoring angiogenesis and metastasis [7, 50, 51].

#### Enabling characteristics

**Fig. 3:**
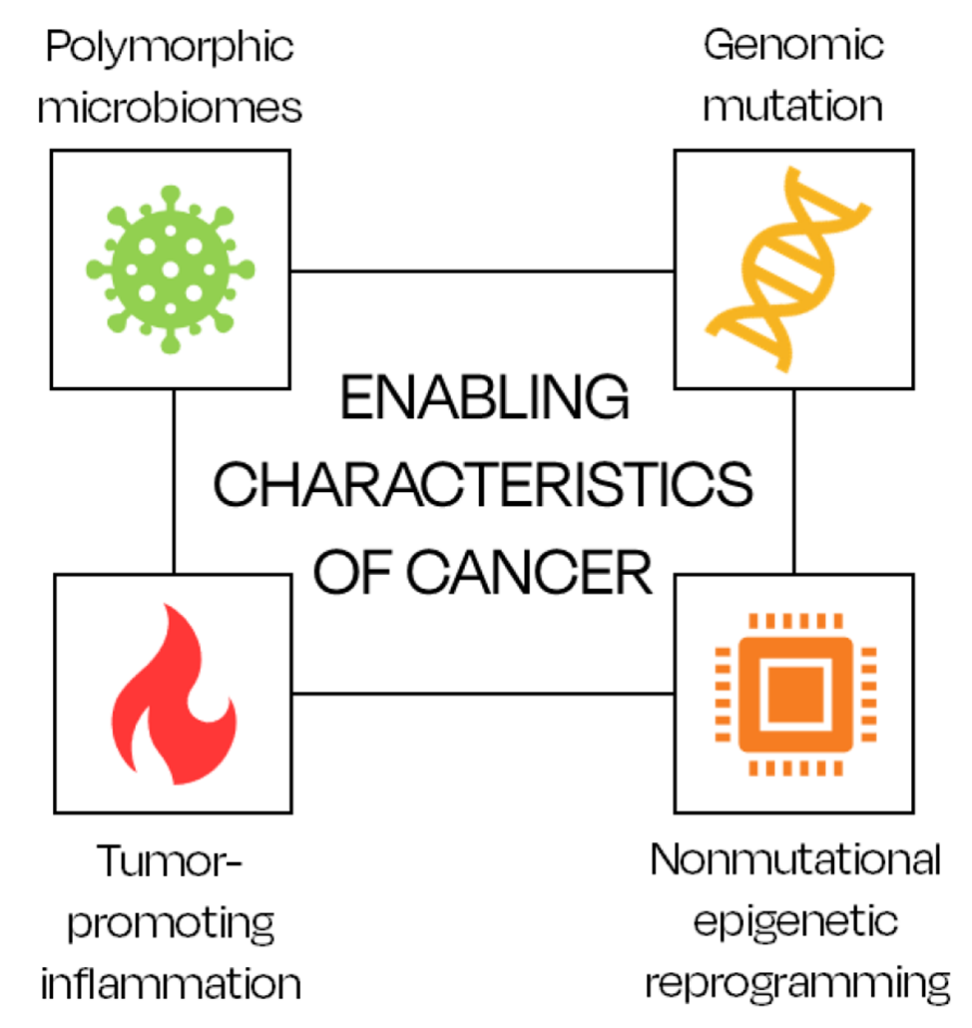
The enabling characteristics of cancer according to Hanahan and Weinberg [5–7]

For the cancer cells to acquire all ten hallmarks (eight confirmed and two additional proposed) described above, Weinberg and Hanahan also addressed four enabling characteristics: genomic mutation, nonmutational epigenetic reprogramming, tumor-promoting inflammation, and polymorphic microbiomes (Fig. 3) [5–7].

Tumor cells may undergo a certain level of genomic mutation [6]; many genetic technologies involve DNA sequencing and are based on individuating genomic mutations to assess their impact on human bodies, from predicting cancer predisposition to evaluating the best mode of treatment for an active malignancy. However, cancer cells can evolve because of pure epigenetically regulated changes in gene expression, which do not involve mutations, hence ‘nonmutational epigenetic reprogramming’ can contribute to the development and progression of cancer, and is currently being assessed for various genetic tests, such as DNA methylation assays or RNA (i.e., ribonucleic acid) sequencing [7, 52].

The inflammatory state is also vital to promote tumor progression. Even though the immune system has always been thought to be a protective tool for the human body, studies have demonstrated how inflammation supplies bioactive molecules to the TME, which are vital to enable the cancer progression [6, 37, 53]. A significant connection with inflammation is given by the role of microbiomes. Polymorphic microbiomes – including those of the gut, lungs, oral cavity, vagina/cervix and skin – are all known to impact the tumor growth, immune evasion ability, genomic instability and therapeutic resistance [7]. The gut microbiome is extremely important for human well-being and has already been proven to influence the incidence and pathogenesis of colon cancer [54].

### The hallmarks of cancer metabolism

Reprogramming energy metabolism is essential for cancer cells to obtain all the nutrients required to fuel their growth and proliferation. Cancer cells have a higher demand for nutrients as they strive to be perpetually active and maintain a continuous proliferative activity, therefore their metabolism needs to change and adapt to sustain the limitations in the TME’s nutrition supply [9]. Along with the hallmarks of cancer, Pavlova and Thompson reviewed the main emerging hallmarks of cancer metabolism in 2016, which were enriched more recently by Pavlova *et al.* in 2022 [8, 9].

**Fig. 4:**
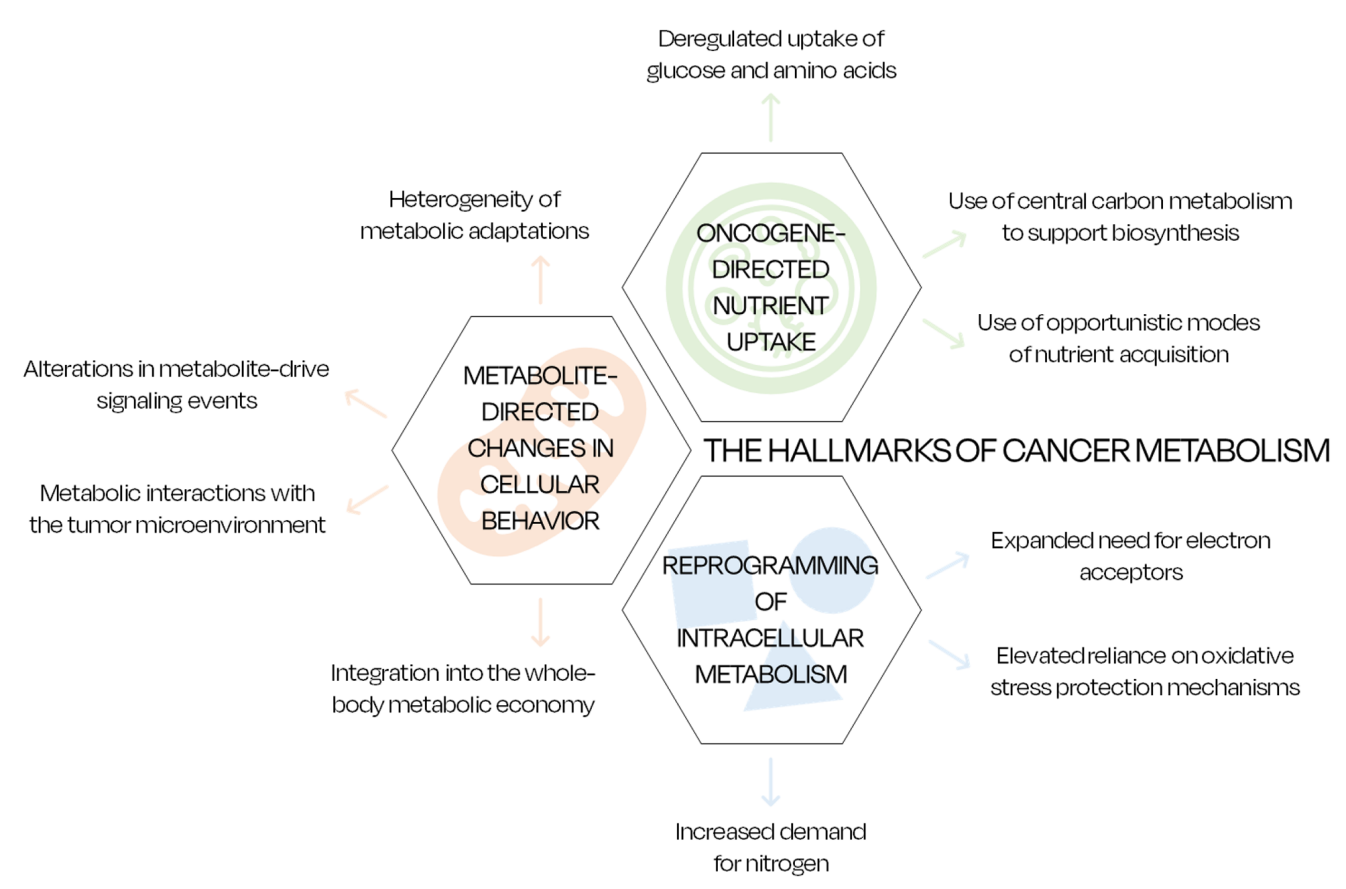
The hallmarks of cancer metabolism according to Pavlova *et al.* [8, 9]

The hallmarks of cancer metabolism can be divided into three main categories. Firstly, the metabolism modifies to receive more nutrients in a physiological process that can be defined as ‘oncogene-directed nutrient uptake’; then, the intracellular metabolism reprograms itself to adapt for the new energetic production; and, lastly, there is an overall impact on the bodily functions caused by metabolite-directed changes in cell behavior (Fig. 4) [8, 9].

### The role of the immune system

The immune system is strictly connected with both cancer development and metabolism. Its role in forming and shaping the immune characteristics of tumor cells is now well-established [55]. The dual nature of the immune system to protect the body from arising malignancies, but also promote tumor progression through inflammation, is defined as ‘cancer immunoediting’ [56]. Immunoediting consists of three main phases. Firstly, the elimination phase (I), when the immune cells successfully destroy developing tumors to guarantee a ‘cancer free’ status to the patient; however, lacking in the destruction of these mutated future-cancer cells is a strong promoter of the equilibrium phase (II), which then utilizes the immune system to maintain cancer cells in a dormant state. Although, due to the great activation of the immune system, other mutations may emerge in this phase which can promote new cancer cell variants. Lastly, the escape phase (III) is entered by cancer cells only when they can no longer be blocked by the immune system; they are also able to induce an immunosuppressive state in the TME [57].

Inflammation is a key process intertwined with cancer immunity. The level of inflammation in the body within cells is extremely important to determine whether the immune system is triggered for protection of their host or active development of cancer. When the immune cascade and a positive feedback are activated by detecting a threat, the original immune system signal is amplified: a tumour cell detected locally can cause increase in immune cell populations and signalling molecules in a systemic way [58]. Acute inflammation, also called ‘innate immunity’, is often the natural step preceding the protective immune responses to both external and internal risk agents (e.g., pathogens and tumor mutations). On the other hand, prolonged inflammation – defined as ‘chronic’ – induces mutational stress favoring cancer initiation, cellular proliferation favoring cancer promotion, and also angiogenesis favoring tissue invasion [57–59]. Both sides of inflammation govern tumorigenesis and the TME, fighting and promoting tumor formation.

There are still many unanswered questions about how the cancer research community can take advantage of the changes to the immune system during pre-tumorigenesis to detect pre-cancerous state, and trace the progress of inflammatory states to understand which vital biomarkers are released in preliminary stages of cancer. For example, there is already research underway that is investigating the detection of advanced adenomas as pre-cancerous condition for colorectal cancers [60, 61], which may benefit from the inclusion of signals relating to immune response rather than solely targeting individual biomarkers.

This extensive summary on oncogenesis describes how complex this mechanism is, from the development of pre-cancerous conditions leading to malignancy to the progression of early developed cancer cells into invasion and the metastatic stage. Current marketed technologies search for specific biomarkers that are characteristic of malignancy; however, given the plethora of mechanisms occurring at each different stage of the neoplasia development, it is utopian to believe that tests designed to target individual markers can alone encompass enough information to provide reliable detection. Cancer is a heterogenous disease, presenting differences not only for the variety of organs affected (e.g., brain, breast, stomach, etc.) but also within the same organ (e.g., for brain: glioblastoma, oligodendroglioma, astrocytoma, etc.). Likewise, during tumorigenesis, there are multiple avenues for tumors to develop, enabling hallmarks to present in a different order and importance. In addition, patient heterogeneity plays its own role in cancer formation and progression, highlighting how focusing on precise classes of biomarkers is limiting the chances of earlier detection. Technologies that examine pan-omic signals and explore the concept of ‘tumor as an organ’, may capture most of the heterogeneity present (i.e., tumor derived and non-tumor derived information) before and during cancer development, and ultimately enable earlier detection.

## A ‘pan-omic’ approach

Since the completion of the Human Genome Project in 2003 allowing the sequencing of the entire human genome (20,000–25,000 genes) [62], various technologies have been developed to understand genetic mutations, including mutations that can lead to cancer. Circulating tumor cells (CTCs), circulating tumor DNA (ctDNA), cell-free DNA (cfDNA), DNA methylation, exosomes, and RNA have all been at the centre of the attention in the last couple of decades for their potential use in cancer detection [63].

Despite the broadening consensus of cancer not being confined to mutating cells but as a complex system interacting with all the bodily surroundings, current ctDNA-based diagnostic methods have shown promising sensitivity and specificity in detecting larger sized tumors; DNA methylation – as shown by Klein *et al*. – is a genetic signature that has been used to discern between distinct types of cancer [64].

ctDNA-based technologies also present drawbacks. For DNA methylation, identifying all the informative methylated CpG fragments contained in the sampled ctDNA, which covers a significant percentage of the genome, still represents an arduous challenge [65–67]. Furthermore, many tumor lesions smaller than 10–15mm in diameter have a mutant allele fraction (MAF; i.e., portion of tumor derived DNA in non-tumor derived DNA) of only 0.01%; a value of 0.01% renders cancer detection a tough task when using a limited 10mL blood draw [68]. In addition, healthy individuals still present with low variable levels of ctDNA in cfDNA, because of other non-cancer somatic mutations or body deterioration due to aging; if these accumulate, they may lead to false positives [68–70]. Ultimately, ct/cfDNA methylation has been linked to the detection of more aggressive cancers with poor prognosis, increasing the prognostic significance of diagnostic tests using this signature but raising doubts about the curability of cancers detected by this method [71].

**Fig. 5:**
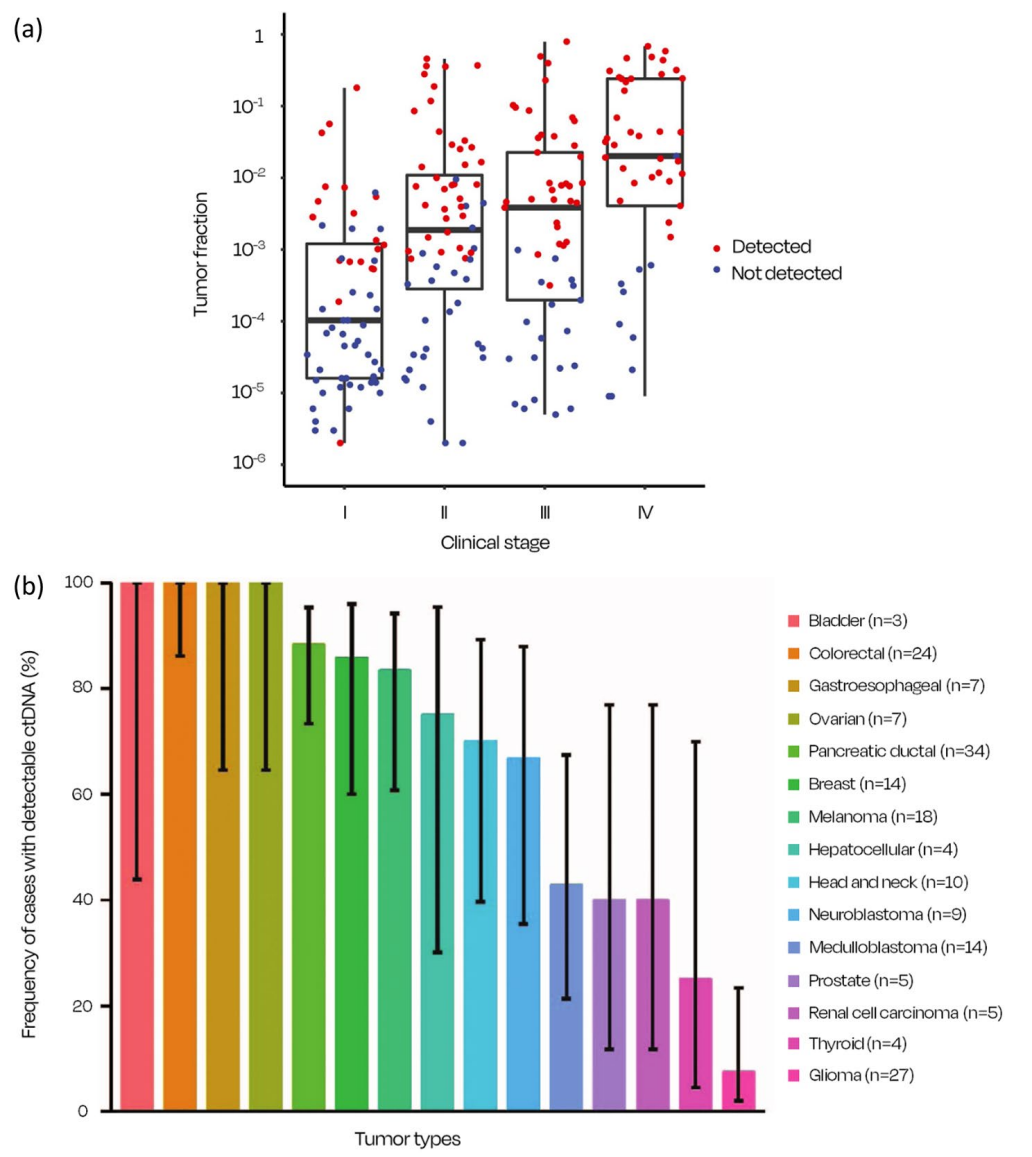
(**a**) Higher circulating tumor allele fraction was observed in later stages of the disease, with increasing cancer detection. Adapted from [75]. (**b**) Fraction of patients with detectable ctDNA in advanced malignances. Adapted from [76]

Current ct/cfDNA tests try to overcome these limitations by tailoring their algorithms for very high specificities (e.g., a fixed 1% false positive rate) [64], but false negatives still represent an issue; low sensitivities are common especially in early stage tumors which only shed minor quantities of cancer material (or not at all) [67, 68, 72–74]. Consequently, there is a negligible detectable signal in ctDNA, in contrast to the amount of material and greater signal in more advanced stages (Fig. 5a); the amount of material varies substantially with the clinical stages of the disease [75]. In addition, different cancers naturally shed different amounts of ctDNA (Fig. 5b) [76]. This results in lack of reliability in recognizing the patients suffering from cancer with low or nonexistent detectable levels of genetic material and varied responses within clinical stages [64, 67, 68, 72–76].

The prohibitive costs of current genetic technologies represent an added limitation for translation of sequencing in the screening/diagnostic stage of health care [68, 69]. Many companies are now assessing multi-omic approaches by combining clinical information in their protocols, such as natural risk factors (e.g., age), specific risk factors (e.g., common biomarkers; for example, prostate specific antigen, PSA, for prostate cancer), and imaging results (e.g., computed tomography, CT; or magnetic resonance imaging, MRI), with their genetic cancer-related biomarkers (e.g., ctDNA; messenger RNA, mRNA; etc.).

A ‘pan-omic’ approach may overcome the limitations of highly specific genetic tests and/or limited biomarker combinations for earlier cancer detection, by assessing cancer as a complex organ and interacting with the entirety of the body through its microenvironment and the immune system. ‘Omics’ have been well established as including genomics, transcriptomics, proteomics, lipidomics, metabolomics, and recently phenomics. Each of these domains potentially contains signals related to the hallmarks of cancer, and the preceding enabling characteristics.

**Fig. 6:**
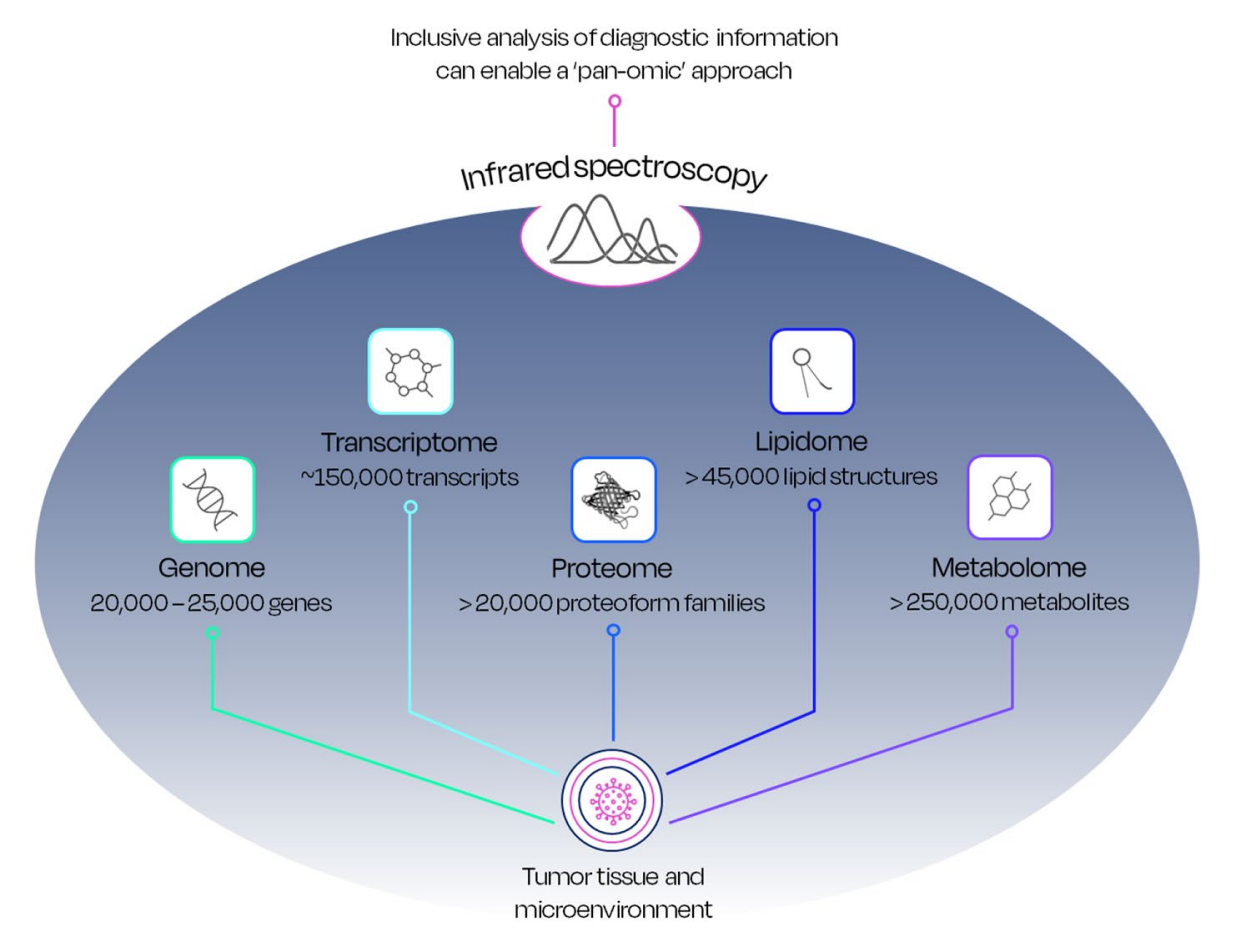
Infrared spectroscopy can enable the ‘pan-omic’ approach by capturing the phenotypic information included in several elements of the whole human body, such as the current -omics; genomics, transcriptomics, proteomics, lipidomics and metabolomics are represented with their relevant units

Encompassing most of the information pertained to cancer development could offer an ideal test, which could still be combined with clinical risk factors, as well as complementary detection methodologies, to fast-track patient referral for cancer diagnosis. Although spectroscopy is limited by the inability of quantifying precise genetic/metabolic information, it is capable of conforming with the ‘pan-omic’ approach thanks to the broad range of spectral (phenotypic) information detectable from the blood, including the TME nature, changes during carcinogenesis, and the corresponding activated immune response (Fig. 6).

### Understanding infrared spectroscopy

**Fig. 7:**
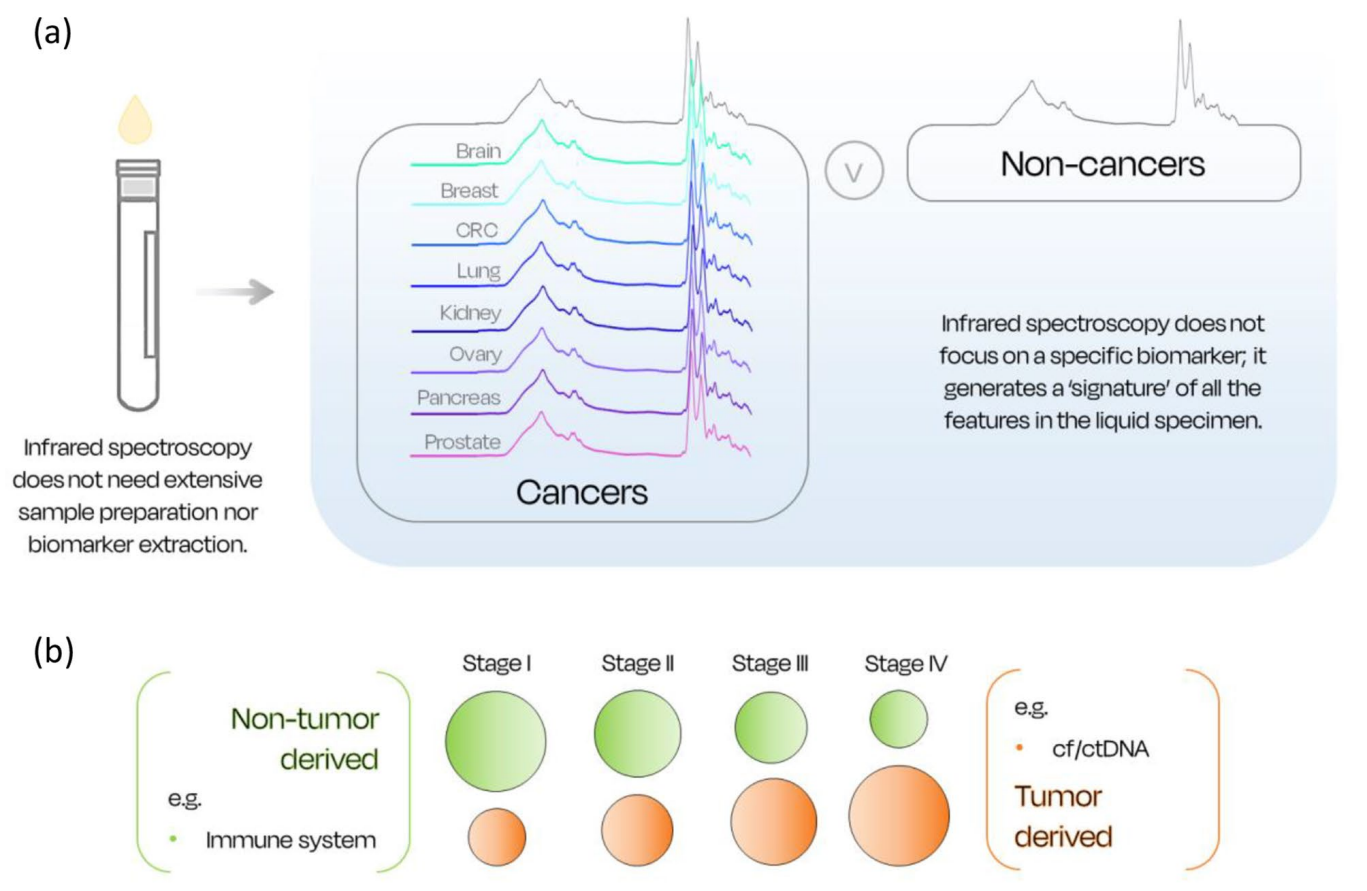
(**a**) Infrared spectroscopy uses unique spectral signatures extracted from the analysis of biofluids to differentiate between diseased and healthy patients. (CRC, colorectal cancer). (**b**) Tumor derived biomarkers (e.g., cf/ctDNA) are more abundant in later stages of the tumor development; whilst non-tumor derived information (e.g., signals from the immune system) are more relevant in early stages and have a greater importance for earlier detection. (cf., cell free; ct, circulating tumor; DNA, deoxyribonucleic acid)

Infrared (IR) spectroscopy is reproducible, non-destructive, easy-to-use, and less expensive than a wide number of medical devices currently used. Moreover, due to technological advances in the field, spectroscopy has attracted great interest for biomedical applications in recent years thanks to the simplicity and minimal invasiveness involved in blood testing [77, 78]. Numerous proof-of-concept studies undertaken in the past decade have successfully demonstrated the effectiveness of clinical spectroscopy for biofluid and tissue specimens [10, 79, 80]. Furthermore, unlike most genetic-based methodologies, there is no need to isolate and extract DNA from samples, which is beneficial in both cost and time (Fig. 7a).

When IR light interacts with a biological sample, the molecular bonds vibrate at different wavelengths, depending on the atoms that compose the molecules in the sample and their related type of bond. After the radiation-sample interaction, the light is directed towards the detector which collects the signature of the sample in the form of a unique spectrum; the differences between cancer and non-cancer patients can be then detected using machine learning algorithms (Fig. 7a).

Blood bathes every organ and cell in the body and provides a unique source of information on the ongoing cellular and extracellular events which span the entire range of biomarker classes. An IR spectrum provides a readout of the full range of this information, embedding both tumor derived and non-tumor derived signals, and including the bodily immune response to the tumor, encompassing the full complexity and heterogeneity of cancer. Non-tumor derived signals, such as the immune response, are higher in earlier stages of the tumorigenesis, where tumor derived signals are lower and hardly detectable (e.g., ct/cfDNA); healthcare needs detection techniques able to capture information from the non-tumor derived signals to enable earlier detection (Fig. 7b).

When detecting differences between cancer and non-cancer samples, the focus is on changes in the macro-compounds within blood of the two classes of patients. For example, differences in amides peaks are related to protein structures, which are key to produce amino acid chains and nucleotides synthesis. Moreover, carbon-oxygen single and double bonds detected in those regions at lower wavenumbers are typical of carbohydrates structures, such as sugars like glucose and its derivatives, which undergo extreme quantitative changes during different phases of tumorigenesis, also given its metabolism implications. Phosphate and lipidic groups changes are also detected; phosphate changes are especially significant as they encompass nucleotides and overall DNA bodily changes, which may include signatures of tumor-related genetic mutations.

The implementation of infrared spectroscopy into the clinical environment still requires large-scale validation studies to achieve robust and accurate diagnostic performance results. The compact design of current spectrometers represents a powerful feature for a non-disruptive implementation into the clinical environment. Hence, spectroscopic liquid biopsies could fit seamlessly in the diagnostic pathways of various diseases since they only require a simple blood draw.

## The emergence of new technologies

There are approximately 150,000 scientific papers documenting thousands of proposed biomarkers, yet only 1% of known markers are routinely used in clinical applications [81]. At present, there are only a few commercialized liquid biopsies currently available, mainly targeted at single-cancer detection rather than multi-cancer detection. The SelectMDx test (MDxHealth, USA) for prostate cancer is a urine-based test and has been successfully launched in the USA and Europe. This test combines the analysis of two mRNA cancer-related biomarkers (HOXC6 and DLX1) and patient’s risk factors (i.e., age, prostate volume, prostate specific antigen levels and digital rectal examination result) [82]. The ExoDx test (Exosome Diagnostics, USA), also for prostate cancer, provides an individual risk score for the patient which determines whether they should be referred for biopsy; it employs exoRNA (i.e., exosomal ribonucleic acid) isolation methodology in a urine sample, RT-qPCR (i.e., quantitative reverse transcription polymerase chain reaction) detection of selected genes (SPDEF, ERG and PCA3) and a specifically developed AI algorithm for the risk score calculation [83, 84]. In addition, the EarlyCDT Lung (Oncimmune, UK) is a blood test released for the triage of patients with an elevated risk of lung cancer into CT scanning; using ELISA (i.e., enzyme-linked immunosorbent assay), it ultimately detects the elevated presence of tumor associated autoantibodies generated by the body’s immune system as a natural defense against cancer cells [85, 86].

The commercial use of these single-cancer liquid biopsies evidences the potential of early detection strategies within healthcare systems; yet, the ability to target more than one type of cancer with only one blood draw could allow even more rapid detection of the disease and enable the most suitable course of treatment for affected patients. Tafazzoli *et al.* investigated the economic potential of a multi-cancer early detection (MCED) genomic blood test complementing current single-cancer screening in the USA, modelling the analysis on a published GRAIL case-control study [87, 88]. The outcomes were promising; improving earlier stages diagnosis, the MCED test could reduce later stage IV diagnoses by 53%, while decreasing costs per cancer treatment by 5,421 USD and increasing quality-adjusted life years (QALY) of 0.13 across all individuals in the screening program and 0.38 across individuals diagnosed with cancer. QALY is a measure of life’s length, weighted by its quality using a standard set of health state quality weights [89]. In addition, Lipscomb *et al.* have highlighted the potential for MCED testing to be cost-effective when building their model on 3 different cancers (pancreatic, uterine and lung) [90]. The development of a robust liquid biopsy capable of detecting multiple cancer types would be ground-breaking and of immense value in the diagnostics field.

### Machine learning algorithms: the promise of AI

There are a few MCED tests currently under investigation, and most of these tests utilize genomic-based methodology to target individual tumor-related biomarkers. Two of these tests are currently in developmental/large-scale testing phase; the Galleri test (GRAIL, USA) and the CancerSEEK liquid biopsy (Exact Sciences Thrive LLC, USA), which both combine blood-based DNA sequencing with machine learning algorithms.

The Galleri test is a liquid biopsy which targets methylation patterns in cfDNA in a patient’s blood to determine the presence of cancer cells [91]. GRAIL indicates that the test can detect more than 50 types of cancer, many of which are not currently screened for. GRAIL’s Galleri test has reported a favorable performance in the diagnosis of more advanced stages – 77% and 91% for stage III and IV, respectively – but detection for early-stage cancer were substantially lower, with only 16% of stage I and 40% of stage II cancers diagnosed successfully [64]. The other test is the CancerSEEK liquid biopsy (Exact Sciences Thrive LLC, USA), which specializes in ctDNA detection followed by the quantification of several cancer-related genomics, transcriptomics and proteomics biomarkers [92, 93]; in their latest retrospectively-assembled case-control feasibility study, Douville *et al.* combined aneuploidy, methylation, protein and mutation markers to enrich detection. It was reported that they can also detect a greater proportion of higher-grade cancers (stage III; 68%, and stage IV; 87%) than early-stage cancers (stage I; 31%, and stage II; 46%) [94].

For an early detection test to be robust and reliable, the technology must be sensitive to earlier stages and have the ability to identify smaller and lower-grade tumors, which in turn would have a greater impact on patient prognosis and survival [74]. The Dxcover^®^ Cancer Liquid Biopsy is a serum-based spectroscopic liquid biopsy which generates a spectral signature that captures the full range of potential markers contained in human blood serum, encompassing both tumor and non-tumor derived signals rather than targeting individual biomarkers. With this approach, 64% of eight different stage I cancers (i.e., brain, breast, colorectal, lung, kidney, ovarian, pancreatic and prostate) were successfully detected at 99% specificity [95]. The technique can be fine-tuned to maximize either sensitivity or specificity depending on the requirements from different healthcare systems and cancer diagnostic pathways, since different intended uses will prioritize different test performance characteristics [96]. This study also tested a symptomatic patient cohort which may be more appropriate in a triage setting, where high sensitivity would be preferred to enable a ‘rule-out’ test. The sensitivity-tuned approach enabled the detection of 93% (214/231) of stage I and 84% (431/516) of stage II cancers, where overall specificity was 61% [95]. This liquid biopsy is not yet available to patients and large-scale clinical validation studies are required to further test its performance.

### Towards combination testing

**Fig. 8:**
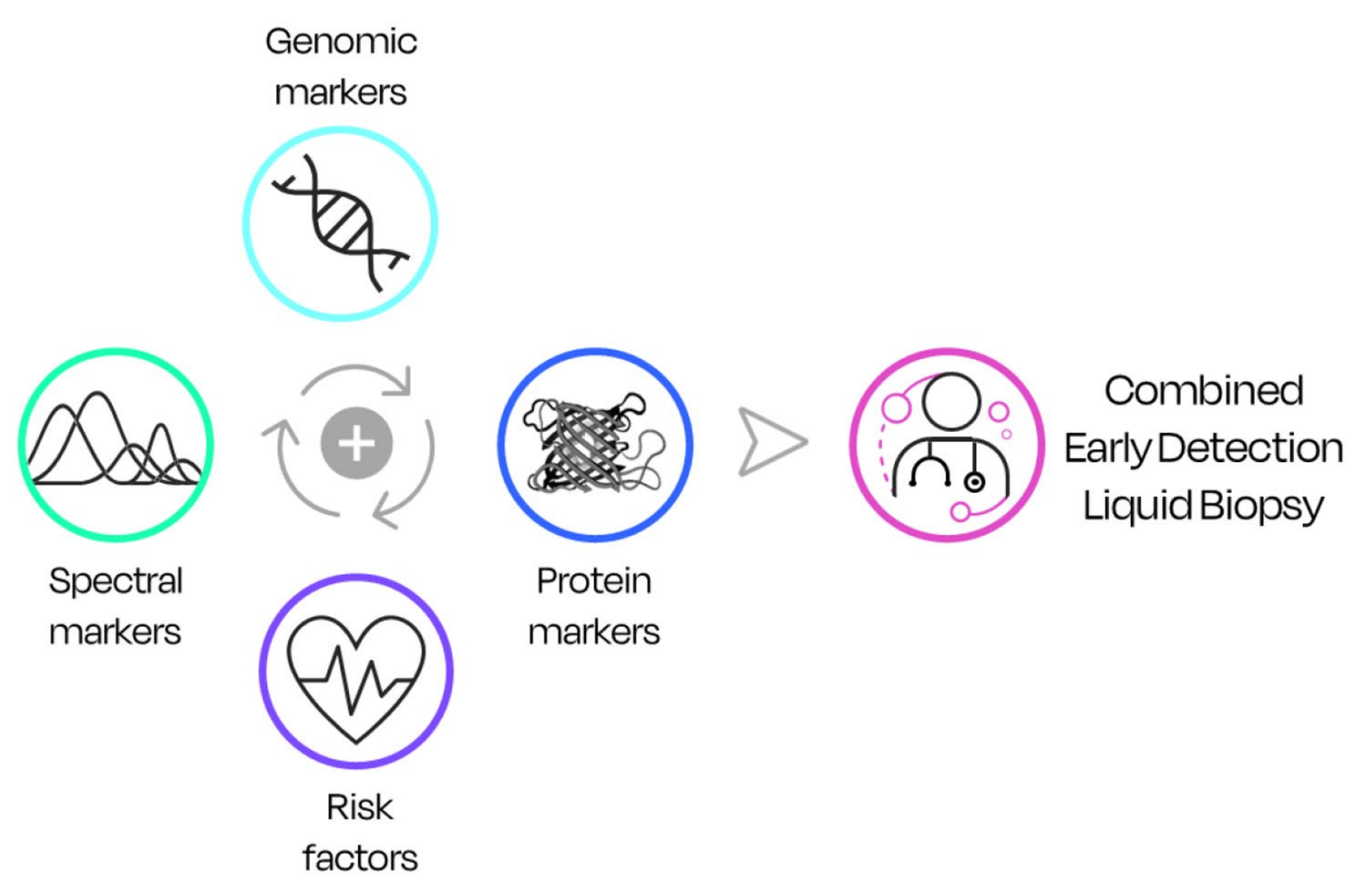
Combined techniques can enable a more accurate early detection liquid biopsy. Adapted from [63]

The future of early detection liquid biopsies is likely to involve contributions from more than one technology platform. Using combinations of techniques that detect diverse types of markers and/or signatures can overcome the issue of low early-stage sensitivity while maintaining an acceptable level of specificity (Fig. 8). Combining approaches may be more fruitful than searching for a ‘winning’ technology.

When considering a screening scenario, various liquid biopsies could be processed simultaneously or sequentially using a single blood draw, without disrupting the clinical pathways. To enhance sensitivity, diagnostic information can be added with the intent that if a positive result is reported for any of these tests, then the patient would be expediated to further diagnostic tests. This can significantly reduce the false negatives; although, to obtain this without compromising the false positive rate, a combined specificity target should be used in test development. Alternatively, affordable, and more sensitive but less specific diagnostic information can be used as a gate-keeper for more expensive and highly specific but less sensitive ones, allowing the control of both cost-effectiveness and budget impact of multi-cancer screening.

Another desirable feature for a combined early detection liquid biopsy would be the ability to accurately predict the tumor origin, which would enable the direction of patients into the most appropriate diagnostic pathway. This would be particularly beneficial both in asymptomatic screening and in cases where patients present with generic non-specific symptoms, when the primary care doctor would prefer to avoid referral for several tests and imaging scans. Combining diagnostic information has the same potential for improving the ability to correctly identify the cancer site as it does for improving detection; different steps can be taken in the process, to prioritize reducing errors for particular types of misclassifications.

## Conclusions

Cancer still represents an incredible global burden. The striving to discover new efficient therapies and techniques to achieve in vivo aid for surgical procedures has been constant over the past few decades, yet detecting cancer earlier may be the answer to improve prognosis, survival rates, and treatment costs. The concept of cancer has evolved throughout the years, changing from being considered solely as mutant cells to a broader concept of ‘tumor as an organ’ [18]. These aggregates of mutant and non-mutant cell types interact with their surroundings through an entire range of metabolic processes, including their involvement with the immune system. Historically, cancer has been viewed as a genetic disease, however oncogenesis depends not only on mutations, but also on several factors arising from non-tumor bodily components/mechanisms, such as the immune system [18–20].

The biochemical, genetic, and metabolic changes that the body undergoes before and during tumorigenesis, to then progress the tumor invasion to other tissues, are vast and complex; the importance of these changes to understand how cancer arises and which factors are key to promote its progression have been heavily investigated [5–9, 57]. Sustaining chronic cell proliferation while avoiding cell growth suppression and cell death, reprogramming cell metabolism to guarantee the necessary fueling uptakes, and exploiting body’s inflammation to induce the immune system to actively participate in promoting angiogenesis, metastasis, and tumor progression, involve several biochemical reactions. That said, the scientific community has yet to identify a precise group of biomarkers that can ascertain pre-cancerous conditions and/or earlier stages of cancer development.

Current marketed liquid biopsies have been focusing on genetic features detectable with genome sequences, limited by the small amount of genetic material shed by certain cancer types and early-stage tumors. Research is still ongoing to obtain more information about pre-cancerous conditions, including immune system changes; however, current technologies have significant associated costs and limit of detection barriers which may be difficult to overcome.

A test that can capture both tumor and immune-related signals would be revolutionary in the diagnostics field. IR spectroscopy would be a promising candidate for a ‘pan-omic’ inclusive approach. The spectral profile of human blood serum encompasses the full complexity and heterogeneity of cancer including tumor and non-tumor derived information, and its potential application for cancer detection has already been highlighted by numerous feasibility studies [10, 79, 80]. The role of AI in cancer detection could also be significant with further development, from fine-tuning the algorithms to maximize either sensitivity or specificity depending on the type of cancer in question and the specific health care requirements, to achieve the ability to accurately predict the tumor origin, which would enable the streamline of patients into the most appropriate diagnostic pathway. Ultimately, collaborations between various companies may enable a combinatorial platform that reliably detects more cancers whilst ruling out those without cancer, promoting a cost-effective combined early detection liquid biopsy.

## Data Availability

No new data were generated or analyzed for this review.

## Abbreviations

AI: artificial intelligence
ATP: adenosine triphosphate
BCAA: branched-chain amino acid
cfDNA: cell-free DNA
CT: computed tomography
CTC: circulating tumor cell
ctDNA: circulating tumor DNA
DLX1: distal-less homeobox 1 (gene)
DNA: deoxy-ribonucleic acid
ELISA: enzyme-linked immunosorbent assay
ERG: ETS related gene
ETS: erythroblast transformation specific (transcription factor family)
exoRNA: exosomal RNA
HOXC6: homeobox C6 (gene)
IR: infrared
MAF: mutant allele fraction
MCED: multi-cancer early detection
MRI: magnetic resonance imaging
PCA3: prostate cancer antigen 3 (gene)
PSA: prostate specific antigen
RB: retinoblastoma-associated protein
RNA: ribonucleic acid
RT-qPCR: quantitative reverse transcription polymerase chain reaction
SAM: sterile alpha motif (protein domain)
SASP: senescence-associated secretory phenotype
SPDEF: SAM pointed domain containing ETS transcription factor
TCA: tricarboxylic acid
TGF-β: transforming grow factorβ
TME: tumor microenvironment
TP53: tumor protein 53
VEGF-A: vascular endothelial growth factor-A
